# Supplementary material for: Associations of Prenatal and Postnatal Maternal Depressive Symptoms with Offspring Cognition and Behavior in Mid-Childhood: A Prospective Cohort Study
Source: Int J Environ Res Public Health. 2019 Mar 20;16(6):1007. doi: 10.3390/ijerph16061007 (PMC6466510; doi:10.3390/ijerph16061007)
Supplement: Supplementary file 1 [file ijerph-16-01007-s001.pdf]

Supplementary Material

Associations of Prenatal and Postnatal Maternal Depressive Symptoms with Offspring Cognition and Behavior in Mid-Childhood: A Prospective Cohort Study

Sabrina Faleschini, Sheryl L. Rifas-Shiman, Henning Tiemeier, Emily Oken and Marie-France Hivert

Table S1. Associations of maternal depressive symptoms in mid-pregnancy with mother-rated behavioral outcomes in mid-childhood (n = 1225).

	Model 1	Model 2<br>β (95% CI)	Model 3
**Behavior and executive function – Mother-rated**			
BRIEF Global Executive Composite	2.60 (0.70, 4.50)	2.02 (0.06, 3.97)	1.97 (0.00, 3.93)
BRIEF Behavior Regulation Index	2.76 (0.90, 4.61)	2.13 (0.22, 4.05)	2.11 (0.18, 4.03)
BRIEF Metacognition Index	2.03 (0.21, 3.86)	1.59 (−0.27, 3.46)	1.54 (−0.34, 3.42)
SDQ Total Difficulties	2.63 (1.60, 3.65)	1.96 (0.92, 2.99)	1.98 (0.95, 3.02)
SDQ Prosocial	−0.21 (-0.57, 0.16)	−0.18 (−0.55, 0.19)	−0.18 (−0.55, 0.20)
SDQ Hyperactivity	0.69 (0.18, 1.19)	0.48 (−0.03, 0.98)	0.47 (−0.03, 0.98)
SDQ Emotional problems	0.75 (0.38, 1.11)	0.55 (0.18, 0.92)	0.56 (0.18, 0.93)
SDQ Conduct problems	0.44 (0.16, 0.73)	0.33 (0.04, 0.62)	0.34 (0.05, 0.63)
SDQ Peer problems	0.75 (0.45, 1.05)	0.60 (0.30, 0.90)	0.61 (0.31, 0.91)

Note. Model 1. Unadjusted; Model 2. Adjusted for maternal race/ethnicity, age at enrollment, education, household income, pre-pregnancy BMI, and smoking during pregnancy and child sex; Model 3. Model 2 additionally adjusted for maternal IQ (KBIT-2).

Table S2. Associations of maternal depressive symptoms at 6 months postpartum with offspring behavioral and cognitive outcomes in mid-childhood in the Project Viva cohort (*n* = 1225).

	Model 1	Model 2	Model 3	Model 4
		β (95% CI)		
**Behavior and executive function**				
**Teacher-rated**				
BRIEF Global Executive Composite	2.78 (0.18, 5.39)	1.11 (−1.49, 3.71)	1.18 (−1.42, 3.78)	0.62 (−2.08, 3.32)
BRIEF Behavior Regulation Index	2.42 (−0.26, 5.10)	1.07 (−1.63, 3.77)	1.05 (−1.65, 3.75)	0.41 (−2.40, 3.22)
BRIEF Metacognition Index	2.86 (0.21, 5.50)	1.20 (−1.44, 3.84)	1.31 (−1.32, 3.95)	0.91 (−1.83, 3.65)
SDQ Total Difficulties	1.46 (0.07, 2.86)	0.74 (−0.62, 2.10)	0.77 (−0.59, 2.13)	0.45 (−0.98, 1.88)
SDQ Prosocial	−0.43 (−0.99, 0.14)	−0.33 (−0.88, 0.22)	−0.34 (−0.89, 0.21)	−0.34 (−0.92, 0.23)
SDQ Hyperactivity	0.46 (−0.23, 1.15)	0.11 (−0.56, 0.77)	0.14 (−0.53, 0.80)	0.03 (−0.66, 0.71)
SDQ Emotional problems	0.32 (−0.11, 0.75)	0.24 (−0.20, 0.68)	0.24 (−0.20, 0.68)	0.13 (−0.33, 0.59)
SDQ Conduct problems	0.45 (0.02, 0.88)	0.25 (−0.18, 0.68)	0.26 (−0.17, 0.69)	0.21 (−0.25, 0.66)
SDQ Peer problems	0.23 (−0.18, 0.63)	0.14 (−0.26, 0.55)	0.14 (−0.27, 0.54)	0.08 (−0.34, 0.50)
**Mother-rated**				
BRIEF Global Executive Composite	1.92 (0.00, 3.84)	1.20 (−0.77, 3.18)	1.15 (−0.83, 3.14)	0.67 (−1.44, 2.78)
BRIEF Behavior Regulation Index	2.52 (0.62, 4.43)	1.95 (−0.01, 3.91)	1.93 (−0.04, 3.90)	1.47 (−0.64, 3.59)
BRIEF Metacognition Index	1.06 (−0.81, 2.92)	0.37 (−1.54, 2.28)	0.31 (−1.60, 2.22)	−0.12 (−2.13, 1.90)
SDQ Total Difficulties	1.47 (0.44, 2.51)	0.63 (−0.41, 1.67)	0.65 (−0.39, 1.69)	0.12 (−0.98, 1.22)
SDQ Prosocial	−0.22 (−0.59, 0.16)	−0.15 (−0.52, 0.23)	−0.15 (−0.52, 0.22)	−0.11 (−0.50, 0.28)
SDQ Hyperactivity	0.16 (−0.35, 0.67)	−0.15 (−0.65, 0.36)	−0.15 (−0.66, 0.35)	−0.30 (−0.84, 0.23)
SDQ Emotional problems	0.61 (0.24, 0.97)	0.42 (0.05, 0.80)	0.43 (0.06, 0.80)	0.30 (−0.09, 0.69)
SDQ Conduct problems	0.13 (−0.14, 0.40)	−0.02 (−0.30, 0.25)	−0.01 (−0.29, 0.26)	−0.11 (−0.41, 0.18)
SDQ Peer problems	0.58 (0.25, 0.90)	0.37 (0.04, 0.70)	0.38 (0.05, 0.71)	0.23 (−0.11, 0.57)
**Cognition**				
KBIT-2 Verbal	−2.83 (−6.32, 0.66)	1.38 (−1.79, 4.54)	0.86 (−2.26, 3.97)	1.32 (−1.99, 4.64)
KBIT-2 Non-verbal	−0.09 (−3.74, 3.56)	2.08 (−1.56, 5.71)	1.70 (−1.90, 5.31)	2.31 (−1.43, 6.06)
WRAVMA Visual-Motor	−2.64 (−6.21, 0.94)	−1.45 (−5.03, 2.13)	−1.64 (−5.23, 1.96)	−1.76 (−5.44, 1.92)
WRAML Design memory	0.31 (−0.29, 0.91)	0.55 (−0.07, 1.16)	0.52 (−0.10, 1.13)	0.62 (−0.02, 1.25)
WRAML Picture memory	0.31 (−0.39, 1.02)	0.52 (−0.21, 1.25)	0.52 (−0.21, 1.25)	0.41 (−0.34, 1.17)
WRAML Visual memory, global score	0.63 (−0.35, 1.60)	1.07 (0.07, 2.07)	1.04 (0.03, 2.04)	1.03 (−0.01, 2.07)

Note. Model 1. Unadjusted; Model 2. Adjusted for maternal race/ethnicity, age at enrollment, education, household income, pre-pregnancy BMI, and smoking during pregnancy and child sex, gestation length, birth weight/gestational age z-score and breastfeeding duration at 6 months; Model 3. Model 2 additionally adjusted for maternal IQ (KBIT-2); Model 4. Model 3 additionally adjusted for mid-pregnancy probable depression.

Table S3. Associations of maternal depressive symptoms at 12 months postpartum with offspring behavioral and cognitive outcomes in mid-childhood in the Project Viva cohort (*n* = 1225).

	Model 1	Model 2	Model 3	Model 4
		β (95% CI)		
**Behavior and executive function**				
**Teacher-rated**				
BRIEF Global Executive Composite	4.39 (1.48, 7.30)	2.05 (−0.84, 4.93)	2.11 (−0.77, 4.99)	1.45 (−1.64, 4.53)
BRIEF Behavior Regulation Index	4.45 (1.49, 7.42)	2.62 (−0.39, 5.63)	2.61 (−0.40, 5.62)	1.99 (−1.28, 5.26)
BRIEF Metacognition Index	3.83 (0.83, 6.83)	1.46 (−1.52, 4.43)	1.56 (−1.42, 4.53)	0.91 (−2.25, 4.07)
SDQ Total Difficulties	2.14 (0.47, 3.81)	1.41 (−0.23, 3.05)	1.45 (−0.19, 3.09)	1.08 (−0.65, 2.82)
SDQ Prosocial	−0.35 (−0.98, 0.28)	−0.32 (−0.96, 0.32)	−0.33 (−0.97, 0.31)	−0.26 (−0.94, 0.41)
SDQ Hyperactivity	0.78 (−0.07, 1.62)	0.47 (−0.34, 1.27)	0.50 (−0.31, 1.30)	0.43 (−0.42, 1.27)
SDQ Emotional problems	0.45 (−0.06, 0.96)	0.33 (−0.19, 0.86)	0.33 (−0.19, 0.86)	0.19 (−0.37, 0.76)
SDQ Conduct problems	0.63 (0.18, 1.08)	0.42 (−0.04, 0.88)	0.42 (−0.04, 0.88)	0.36 (−0.13, 0.84)
SDQ Peer problems	0.29 (−0.19, 0.76)	0.20 (−0.29, 0.69)	0.19 (−0.30, 0.68)	0.11 (−0.43, 0.65)
**Mother-rated**				
BRIEF Global Executive Composite	2.96 (0.83, 5.09)	2.32 (0.10, 4.55)	2.29 (0.06, 4.51)	1.79 (−0.60, 4.18)
BRIEF Behavior Regulation Index	3.38 (1.35, 5.42)	2.85 (0.73, 4.97)	2.84 (0.72, 4.97)	2.17 (−0.11, 4.46)
BRIEF Metacognition Index	2.03 (−0.03, 4.09)	1.50 (−0.65, 3.66)	1.46 (-0.70, 3.61)	1.15 (−1.15, 3.46)
SDQ Total Difficulties	2.56 (1.39, 3.73)	1.69 (0.51, 2.88)	1.71 (0.53, 2.89)	1.20 (−0.10, 2.50)
SDQ Prosocial	−0.13 (−0.53, 0.27)	−0.13 (−0.54, 0.27)	−0.13 (−0.54, 0.27)	−0.03 (−0.47, 0.41)
SDQ Hyperactivity	0.84 (0.27, 1.40)	0.57 (0.00, 1.13)	0.56 (−0.01, 1.13)	0.53 (−0.08, 1.15)
SDQ Emotional problems	0.74 (0.33, 1.14)	0.52 (0.10, 0.94)	0.53 (0.11, 0.95)	0.35 (−0.11, 0.81)
SDQ Conduct problems	0.45 (0.14, 0.77)	0.32 (−0.01, 0.65)	0.33 (0.00, 0.66)	0.27 (−0.09, 0.63)
SDQ Peer problems	0.54 (0.19, 0.89)	0.29 (−0.07, 0.65)	0.30 (−0.06, 0.66)	0.05 (−0.33, 0.44)
**Cognition**				
KBIT-2 Verbal	−7.59 (−11.6, −3.61)	−1.56 (−5.09, 1.97)	−2.00 (−5.49, 1.49)	−2.21 (−5.95, 1.54)
KBIT-2 Non-verbal	−3.02 (−7.13, 1.10)	0.20 (−3.92, 4.32)	−0.12 (−4.19, 3.96)	0.12 (−4.30, 4.55)
WRAVMA Visual-Motor	−3.73 (−7.87, 0.41)	−2.56 (−6.83, 1.71)	−2.72 (−6.99, 1.55)	−2.60 (−7.27, 2.08)
WRAML Design memory	−0.26 (−0.96, 0.43)	0.00 (−0.71, 0.71)	−0.03 (−0.74, 0.68)	−0.12 (−0.88, 0.63)
WRAML Picture memory	−0.64 (−1.41, 0.13)	−0.53 (−1.32, 0.27)	−0.53 (−1.33, 0.26)	−0.93 (−1.79,-0.08)
WRAML Visual memory, global score	−0.90 (−2.02, 0.21)	−0.53 (−1.66, 0.61)	−0.56 (−1.70, 0.57)	−1.06 (−2.27, 0.16)

Note. Model 1. Unadjusted; Model 2. Adjusted for maternal race/ethnicity, age at enrollment, education, household income, pre-pregnancy BMI, and smoking during pregnancy and child sex, gestation length, birth weight/gestational age z-score and breastfeeding duration at 12 months; Model 3. Model 2 additionally adjusted for maternal IQ (KBIT-2); Model 4. Model 3 additionally adjusted for probable depression in mid-pregnancy and 6 months postpartum.
